# A TaqMan real-time PCR method based on alternative oxidase genes for detection of plant species in animal feed samples

**DOI:** 10.1371/journal.pone.0190668

**Published:** 2018-01-02

**Authors:** Maria Doroteia Campos, Vera Valadas, Catarina Campos, Laura Morello, Luca Braglia, Diego Breviario, Hélia G. Cardoso

**Affiliations:** 1 ICAAM—Instituto de Ciências Agrárias e Ambientais Mediterrânicas, Universidade de Évora, Pólo da Mitra, Évora, Portugal; 2 Istituto Biologia e Biotecnologia Agraria, Milan, Italy; Western Australia Department of Agriculture and Food, AUSTRALIA

## Abstract

Traceability of processed food and feed products has been gaining importance due to the impact that those products can have on human/animal health and to the associated economic and legal concerns, often related to adulterations and frauds as it can be the case for meat and milk. Despite mandatory traceability requirements for the analysis of feed composition, few reliable and accurate methods are presently available to enforce the legislative frame and allow the authentication of animal feeds. In this study, nine sensitive and species-specific real-time PCR TaqMan MGB assays are described for plant species detection in animal feed samples. The method is based on selective real-time qPCR (RT-qPCR) amplification of target genes belonging to the alternative oxidase (*AOX*) gene family. The plant species selected for detection in feed samples were wheat, maize, barley, soybean, rice and sunflower as common components of feeds, and cotton, flax and peanut as possible undesirable contaminants. The obtained results were compared with end-point PCR methodology. The applicability of the AOX TaqMan assays was evaluated through the screening of commercial feed samples, and by the analysis of plant mixtures with known composition. The RT-qPCR methodology allowed the detection of the most abundant species in feeds but also the identification of contaminant species present in lower amounts, down to 1% w/w. AOX-based methodology provides a suitable molecular marker approach to ascertain plant species composition of animal feed samples, thus supporting feed control and enforcement of the feed sector and animal production.

## Introduction

In the last few years, the traceability and labelling of processed food and feeds has gained more attention, especially in the cases where the identification of different components is difficult or even impossible [1–3]. Ruminant feeds are complex mixtures of raw and processed plant materials and industrial by-products, not easily recognizable by naked eye. In spite of the existence of mandatory traceability requirements for the analysis of feed composition, addressed by Regulation (EC) 767/2009 [4], labels do not always provide sufficient guarantee about the real species and additives’ composition of those products [3]. Besides the economical impact of the fraud, the absence of species described in the label or the presence of unknown species could compromise the quality of the final product, since it is well known that animal diet is regarded as determinant for both animal health and product quality [5,6]. In fact, the alteration on milk flavour and other organoleptic characteristics can consequently affect the quality of the final product [7]. This has more economic impact in case of products with Protected Designation of Origin (PDO). Species verification in feeds is a legal traceability requirement and has interest on the side of feed sector and on the side of animal producers as the ones that buy the feeds and want to ensure the quality they give to their animals. Because PDO (Protected Designated of Origin) productions are often associated to restrictions in the use of specific plant species in the feed, to guarantee its correct labelling becomes even more important. Until now, the traditional method to ascertain the botanical identity of raw materials and the composition of processed material or complex feed mixtures relies on mesh fractionation steps followed by analysis under the optical microscope, with botanical species recognized on the base of particular plant tissues and structures. Consequently, there is a need for more reliable, accurate and powerful analytical techniques to enforce the legislative frame and allow the authentication of animal feeds as complex mixtures of different plant materials. Efficient methodologies for species authentication in processed foods and feeds, following different molecular approaches have been developed in the last few years [8]. However, very few reports, relying on both end-point and Real-time PCR, describe the use of those techniques to detect plant species in feed samples [9–11]. Real-time PCR instrumentation requires considerably short hands-on time, and detection of amplified products is automated, simple and reproducible [12]. When combined with the chemistry of TaqMan MGB probes, this technique represents the most specific and sensitive detection system if few target DNA is present. Braglia and co-authors [11] reported the most accurate known method that allowed plant species detection in feed samples, based on species-specific TaqMan MGB assays designed on Tubulin gene family members. This method, called Tubulin-Based-Polymorphism (TBP), initially based in amplicons separation in acrylamide gels [10] has been improved in the frame of an EU funded project named Feed-Code (http://www.feedcode-project.eu).

In the frame of the same EU project, a method based on the alternative oxidase (AOX) gene family, initially proposed as end-point methodology for validation of the TBP method [13] is here presented as a reliable molecular marker approach to ascertain plant species composition of animal feed samples using species-specific TaqMan assays. AOX is a nuclear encoded inner mitochondrial membrane protein that has been described in all plants as terminal oxidase in the alternative (cyanide-resistant) respiration pathway. In higher plants, AOX is encoded by a small multigene family with members distributed in two subfamilies: AOX1 and AOX2-type. Members of AOX1-subfamily have been described in monocots and dicot plant species, while the presence of AOX2-subfamily members has been reported only in dicot plant species [14]. High variability has been reported in *AOX* genes [14,15], especially at intron level [16–19], which allows to differentiate among plant species. In this study we describe the development and testing of nine TaqMan MGB assays, based on selective RT-qPCR amplification of target genes belonging to the *AOX* gene family for specific plant species identification in animal feed samples. Results were compared with end-point methodology. The target species were selected amongst the most common plant species used as components of feeds, or by the contrary, the ones considered as feed contaminants due to the negative effect that they might have on the quality of the final product (milk and cheese). In the specific case of products adopting PDO or PGI (Protected Geographical Indication) denomination, is even more important to ensure authenticity and counteract events (or charges) of product adulteration. The applicability of the AOX TaqMan assay was evaluated through the screening of commercial feed samples, and confirmed by the analysis of plant mixtures with known composition.

## Materials and methods

### Raw materials, mixtures and feeds

Plant raw materials, mixtures of raw materials and feed samples were used for detection of nine plant species (Table 1). Raw materials consisted in flour or in intact seeds of 31 plant species provided separately and properly identified by the Feed-Code partners (see S1 Table). Mixtures of plant raw materials and feed samples consisted in ground fine powder (also sent by the Feed-Code partners).

**Table 1:** List of the target species included in the study.

Common name	Scientific name
Maize	*Zea mays*
Barley	*Hordeum vulgare*
Soybean	*Glycine max*
Wheat	*Triticum spp*
Flax	*Linum usitatissimum*
Sunflower	*Helianthus annuus*
Peanut	*Arachis hypogaea*
Rice	*Oryza sativa*
Cotton	*Gossypium hirsutum*

Three groups of samples were tested:

1^st^ group: 2 reference mixtures (RM) of feed raw materials mimicking the composition of commercial feeds. The composition of each mixture, with the respective amount of each plant species is included in Table 2.

2^nd^ group: 8 feed samples with labeled species included (7 of them sent by the Feed-Code partners and 1 commercial cow feed).

3^rd^ group: 7 mixtures composed by 1 to 8 different raw materials, in different combinations, for an inter-laboratory comparison (ILC) procedure, assigned to the participants by Eurofins Food and Feed Testing, Norway AS (http://www.eurofins.com/food-and-feed-testing/). The plant species considered for this assay were wheat, barley, soybean, maize, cotton, sunflower, rice and flax.

**Table 2:** Reference mixtures (RM) with known composition, and respective percentage in a total of 100 g.

RM 1	%	RM 2	%
Maize	20	Maize	15
Barley	20	Barley	15
Soybean	20	Soybean	15
Wheat (durum+soft)	20	Wheat (durum+soft)	15
Sugar beet	6	Sugar beet	10
Flax	4	Flax	8
Sunflower	6	Sunflower	10
Peanut	1	Peanut	3
Rice	1	Rice	3
Cotton	1	Cotton	3
Rapeseed	1	Rapeseed	3

### Genomic DNA (gDNA) extraction and analysis

In order to assess primers/probe specificity, gDNA was extracted from the 31 plant species included in the Feed-Code project (see plant species list in S1 Table). Seed samples were ground to a fine powder using liquid nitrogen. From each sample, 50–100 mg were used for gDNA extraction with the Maxwell 16 Tissue DNA Purification kit (Promega, Madison, USA), in the Maxwell 16 Instrument (Promega, Madison, USA). The same procedure was followed for the different samples (raw material mixtures and feeds). To quantify and assess gDNA purity, the absorbance was measured on a NanoDrop-2000C spectrophotometer (Thermo Scientific, Wilmington, DE, USA). gDNA integrity was checked by 0.8% agarose gel electrophoresis and visualized using GeneTools (Syngene, Cambridge, UK) after staining with EtBr solution (2 ngmL^-1^). Samples were diluted to a concentration of 10 ngμL^-1^ to be used in cross-reactivity tests and of 20 ngμL^-1^ for all the remaining analyses.

### AOX end-point PCR methodology

*AOX* gene sequences from soybean were retrieved by BLASTn search into the freely available NCBI database (http://www.ncbi.nlm.nih.gov). Sequences of maize and rice were retrieved from Plaza 3.0 (http://bioinformatics.psb.ugent.be/plaza/versions/plaza_v3_monocots/). Sequence from barley was retrieved from the IPK Barley Blast Server (http://webblast.ipk-gatersleben.de/barley/) and sequence of wheat was from e!EnsemblPlants (http://plants.ensembl.org/Multi/Search/New?db=core). Since *AOX* sequences from cotton, sunflower, flax and peanut were not available in public databases, the isolation of partial sequences was previously performed, and sequences were published in NCBI databases (Table 3).

**Table 3:** Sequences of RT-qPCR oligonucleotide primers and probes designed on *AOX* gene sequences. AS: amplicon size.

Species	Accession ID	Primers (5’→ 3’)	Probes (5’→ 3’)	As(bp)
Maize	ZM05G37570	Fw: GCCCTCTGAGTTCCTTTGCTT Rv: GAGCTGCATACCCTGGAAATG	TGATGCGCAGGACAT	92
Barley	CAJW010038523	Fw: TTGATTCTGAACTCCTGACGATGT Rv: CACGTCCTGCACGACACAAT	TGCTGATCTGGTCTGTC	63
Soybean	NM_001248837.1	Fw: ATGAAGCTCACTGCACTGAATTCT Rv: GGCGGTTACCGTTTTGGTT	CTGCTCAATGGCC	73
Wheat	TRAES3BF079200040CFD	Fw: GCTACCACTGAGGAAAGGGACTAC Rv: TGCATCCAAACAAGCTCCAA	CCGTACGCAATAAA	63
Flax	MF281398	Fw: TCGCATTCTGGTCTGTCAAATC Rv: AAGGGAGTAACCTGAAAGAACAAGTC	CTCCGCTGGCCAAC	64
Sunflower	MF281399	Fw: TCCAGCACCTGTGGGTTACC Rv: ACCCATACCACCAGAATCCTATACC	AAACCGTTTGCTTATAGAGG	69
Peanut	MF281397	Fw: TTTTTTGGAAGATCTGGTCCAGTT Rv: TACGAGGCAAAAAATCAGGTACAG	CTTAACCTAAAGTTTCAGCTGAT	128
Rice	OSINDICA_02G20320	Fw: ATCGCCATTAAGGGATCAATAAATA Rv: CATTGCATGCGTCCAAATAATAC	CCGGAGACAATAAT	67
Cotton	MF281396	Fw: GACGGCTCCCCATGGAA Rv: TGTTGCACGAAAAAGACCAATT	TGGACCTGCTTTAGGG	63

AOX species-specific primers were designed based on genomic sequences of *AOX* gene members belonging to both *AOX1* and *AOX2*-subfamilies, considering for that the intronic and UTR regions due to the high variability detected across-species. PCR optimization was made at species level. Primers sequences are described by [13] and PCRs conditions are here summarized. PCRs were carried out for 35 cycles in a T100^TM^ Thermal Cycler (Bio-Rad) and the GoTaq polymerase (Promega, Madison, WI, USA) was used following manufacturer’s instruction. Each cycle consisted in a denaturation step for 30 s at 94°C followed by the annealing step for 30 s that occurred at variable temperatures depending on the species and the extension step at 72°C [13]. An initial step at 94°C for 5 min and a final step at 72°C for 10 min were also considered. PCR products were separated in 2% (w/v) agarose gel and subsequently analyzed, after Ethidium Bromide staining (2 ng mL^-1^) on a Gene Flash Bio Imaging system (Syngene, Cambridge, UK). Each plant species is characterized by a single specific DNA fragment, ranging from 180 to 400 bp. The fragment was previously confirmed as the target AOX gene by cloning and sequence analysis. Peanut was additionally included in the end-point analysis (primer forward: 5’-AAGCATCTTGCAACAGGACA-3’, primer reverse: 5’-TCTACCAAACAATCATCAAATCA-3’; annealing step at 58°C and 3.0 mM of MgCl_2_).

### Design of species-specific TaqMan assays and RT-qPCR conditions

Primers and probes were designed for the nine plant species using the Primer Express 3.0 software for Real-Time PCR (Applied Biosystems Foster City, CA, USA) (Table 3), selecting the option MGB TaqMan probes with amplicon length 63 to 128 bp, and using the default parameters of the software. As target for design of the TaqMan assays we considered the sequences generated by the primers used in the AOX end-point PCR methodology. To avoid the existence of false negatives, a bioinformatic approach was performed to assure that primers and probes were specific at species level through the alignment of sequences from different cultivars. RT-qPCRs were carried out on a 7500 Real Time PCR System (Applied Biosystems, Foster City, CA, USA) using 80 ng of gDNA as template, 2x Luminaris Color Probe Low ROX qPCR Master Mix (ThermoFisher Scientific) and 20x TaqMan Gene Expression Assays (Applied Biosystems) in a total volume of 20 μL. The quantification cycle (Cq) values were acquired for each sample with the Applied Biosystems 7500 software (Applied Biosystems, Foster City, CA, USA), with the following cycling conditions: 10 min at 95°C for initial denaturation, an amplification program of 40 cycles at 95°C for 15 s and 60°C for 1 min. The fluorescence threshold was manually set above the background level. Two gDNA extractions and two technical replicates were analyzed for each sample. Positive target controls (gDNA of each plant species) and no template controls were included in all plates.

### DNA calibrator plasmids

In order to produce a set of calibrator plasmids to be used on RT-qPCR, the Taqman target regions were amplified using the previously described end-point species-specific primers. Amplicons, with sizes ranging between 202 and 983 bp, were cloned into a pGem®-T Easy vector (Promega, Madison, WI, USA) and used to transform *E*. *coli* JM109 (Promega Madison, WI, USA) competent cells, by standard procedures. Recombinant plasmid DNAs were extracted by alkaline lysis protocol [20], and concentration of purified plasmid DNA was determined in a NanoDrop-2000C spectrophotometer (Thermo Scientific, Wilmington, DE, USA). Plasmid inserts were verified by commercial sequencing using T7 and SP6 universal primers (Macrogen company: https://dna.macrogen.com/eng/).

## Results and discussion

### TaqMan assay specificity

To confirm the specificity, each probe was tested for cross-reactivity in gDNA of 31 plant species, bulked into seven pools (for information on the species and composition of the pools see S1 Table and S2 Table), containing gDNA at 10 ngμL^-1^ for each component. Any of the species-specific probes successfully and selectively targeted its correspondent gDNA. No cross-reaction with other species was found (S1 Fig).

### Calibration curves and probe validation

An absolute DNA quantification method, based on the determination of the absolute number of target copies (TCN) present in a given sample, corresponding to haploid genome equivalents, was chosen for our assays. This approach relies on external calibration performed with samples of known target copy number. In order to obtain a specific calibrator, a DNA region that included the corresponding TaqMan target region was amplified by end-point PCR using *AOX* species-specific primers, cloned into a plasmid vector, and further used to establish a calibration curve using the respective TaqMan qPCR assay. Ten-fold dilution series were used to draw seven-point calibration curves to validate the probes in the dynamic range chosen for the assay (8E1 to 8E7 target copies). The correlation between measured Cq values and the logarithm of the concentration was determined by linear regression analysis.

Amplification efficiency, calculated with the equation E = (10^(−1/slope)^ − 1) × 100, slope and linearity (coefficient of determination, R^2^) for each probe were calculated. Amplification efficiency ranged between 90 and 110%, slope between -3.723 and -3.147 and R^2^ = 0.99 (Table 4).

**Table 4:** Efficiency and precision of the RT-qPCR assays in the nine plant species.

Target	Efficiency (%)	Slope	R^2^
Maize	100	-3.314	0.991
Barley	108	-3.147	0.994
Soybean	96	-3.412	0.998
Wheat	92	-3.723	0.999
Flax	94	-3.477	0.999
Sunflower	90	-3.596	0.998
Peanut	97	-3.398	0.994
Rice	104	-3.226	0.997
Cotton	98	-3.369	0.999

Almost all parameters fell within the acceptance criteria, E = 90–110%, −3.6≤slope≤−3.1 and R^2^>0.98 (Table 4), set by International Institutions for both GMO detection done by RT-qPCR [21] and for the quantification of specific DNA sequences in food (http://gmo-crl.jrc.ec.europa.eu/doc/Min_Perf_ Requirements_Analytical_methods.pdf and Codex Committee on Methods of Analysis and Sampling. CAC/GL 74–2010).

### Assay of sensitivity and linearity on raw materials

To evaluate the sensitivity and linearity of the species-specific RT-qPCR systems, dilution series of gDNA extracted from the nine target plant species were used. gDNA dilutions ranged from final concentration of 0.04 ng to 80 ng, in a total of nine data points. Dilution series were made in a mixture of gDNA solutions that excluded the species under study. Standard curves were automatically generated by the instrument software (Applied Biosystems, Foster City, CA, USA). Amplification plots are indicated in S2 Fig. All species presented a linear correlation (R^2^>0.98) between TCN and template gDNA amount in the whole concentration range (S3 Fig), confirming the reliability of the assay and suggesting absence of PCR inhibitors.

For each species, Cq values between 34 and 35 were taken as indicative of traces amounts, while a Cq = 35 was considered the cut off limit, defining no detection. In accordance, the lowest amounts that could be detected for each species and respective Cq values and TCN are indicated in Table 5.

**Table 5:** Lowest amount of detection of each plant species by RT-qPCR assay based on *AOX* genes. TCN: target copy number.

Target	Lowest amount	Cq	TCN
Maize	0.04 ng	32.1	55.4
Barley	0.04 ng	34.3	8.4
Soybean	0.04 ng	33.5	73.8
Wheat	0.2 ng	33.5	159.7
Flax	0.2 ng	34.9	28.7
Sunflower	0.04 ng	34.3	377.3
Peanut	0.4 ng	34.0	2003.1
Rice	0.04 ng	34.4	8.8
Cotton	0.04 ng	32.9	65.0

### TCN80 absolute quantification in mixtures of known composition

To evaluate target gDNA amount in terms of TCN using 80 ng of gDNA (TCN80), two flour mixtures of known botanical compositions (RM1 and RM2) were analyzed (Table 6). The nine qPCR assays were tested on duplicate gDNA extractions (with two technical replicates performed). As expected, for each target species, higher TCN80 values were observed with correspondingly higher w/w % values.

**Table 6:** qPCR assay based on *AOX* genes applied to reference samples. RM: reference mixture; TCN80: target copy number using 80 ng of gDNA (mean of two replicates).

Target	Sample	w/w %	Cq	TCN80
Maize	RM1	20	27.52	1327.5
	RM2	15	28.24	802.8
Barley	RM1	20	26.91	1859.5
	RM2	15	27.60	1113.7
Soybean	RM1	20	29.69	680.3
	RM2	15	29.80	623.8
Wheat	RM1	20	27.79	5542.3
	RM2	15	28.25	4195.6
Flax	RM1	4	26.87	6275.6
	RM2	8	25.59	15491.5
Sunflower	RM1	6	28.05	19089.6
	RM2	10	27.52	24564.2
Peanut	RM1	1	34.27	1939.2
	RM2	3	33.86	2581.0
Rice	RM1	1	33.89	13.5
	RM2	3	31.89	55.8
Cotton	RM1	1	28.75	1108.7
	RM2	3	27.24	3029.2

The qPCR methodology in mixtures of plant species of known composition allowed the detection of species that are usually abundant in feeds such as wheat, barley, soybean and maize, as well as the detection of species present in lower amounts, down to 1% w/w, which is considered as an unwanted contaminant level in some specific cases.

### Analysis on feed samples

TaqMan assays designed on *AOX* genes were tested on a group of dairy cow feed samples (7 samples provided by the Feed-Code project partners plus an additional commercial feed) with declared species composition included in the label. The results were compared with the AOX based end-point PCR methodology. Table 7 reports the results of the analysis.

**Table 7:** Feed analyses for conformity to the label. Results using Taqman assay and end-point PCR methodology based on *AOX* genes. TCN80: target copy number using 80 ng of gDNA; D: detected; ND: not detected; NT: not tested.

Feed	Species included in the label	AOX TaqMan probes			AOX end-point probes (D/traces/ND)
		Species identified	Cq value	TCN80	
CPR-X8	Maize	Maize	27.92	1025.6	Maize (D)
	Soybean	Soybean	29.03	1067.6	Soybean (D)
	Barley	Barley	31.38	70.9	Barley (D)
		Wheat	31.40	611.6	Wheat (D)
		Sunflower	34.28	394.3	Sunflower (traces)
		Rice	34.21	10.7	Rice (ND)
CPR-X10	Maize	Maize	28.06	957.4	Maize (D)
	Soybean	Soybean	28.91	1179.8	Soybean (D)
	Flax	Flax	>35	ND	Flax (ND)
		Wheat	28.36	4001.4	Wheat (D)
		Sunflower	30.91	3070.9	Sunflower (D)
		Barley	>35	ND	Barley (traces)
CPR-X13	Maize	Maize	28.26	791.2	Maize (D)
	Wheat	Wheat	28.47	3626.6	Wheat (D)
	Soybean	Soybean	29.88	592.9	Soybean (D)
	Barley	Barley	31.29	75.4	Barley (D)
	Sunflower	Sunflower	>35	ND	Sunflower (ND)
CPR-X32	Maize	Maize	29.08	457.8	Maize (D)
	Barley	Barley	30.28	157.5	Barley (D)
	Soybean	Soybean	32.13	130.7	Soybean (traces)
	Durum wheat	Wheat	27.69	5893.7	Wheat (D)
	Soft wheat				
	Sunflower	Sunflower	32.41	1205.6	Sunflower (traces)
	Sugarbeet	No probe	-	-	Sugarbeet (ND)
CPR-X42	Maize	Maize	29.11	445.3	Maize (D)
	Barley	Barley	34.41	7.8	Barley (traces)
	Wheat	Wheat	28.33	4005.5	Wheat (D)
	Soybean	Soybean	33.18	64.1	Soybean (traces)
		Rice	27.31	1444.4	Rice (D)
		Sunflower	>35	ND	Sunflower (traces)
CPR-z2	Maize	Maize	27.64	1293.8	Maize (D)
	Soybean	Soybean	31.12	256.0	Soybean (D)
	Barley	Barley	30.53	135.0	Barley (D)
	Wheat	Wheat	29.85	1550.2	Wheat (D)
	Sunflower	Sunflower	32.68	1031.2	Sunflower (traces)
	Sugarbeet	No probe	-	-	Sugarbeet (ND)
CPR-z3	Soybean	Soybean	28.01	2172.9	Soybean (D)
	Sunflower	Sunflower	30.21	4703.2	Sunflower (D)
	Wheat	Wheat	28.10	4833.6	Wheat (D)
	Maize	Maize	31.23	102.0	Maize (traces)
		Barley	33.20	19.6	Barley (traces)
		Flax	33.93	68.4	Flax (ND)
		Rice	34.22	10.7	Rice (ND)
PT1	Barley	Barley	29.61	256.3	NT
	Maize	Maize	28.65	607.7	NT
	Rice	Rice	25.81	4253.9	NT
	Soybean	Soybean	30.92	299.5	NT
	Sunflower	Sunflower	32.51	1137.9	NT
	Wheat	Wheat	29.41	2036.7	NT
	Rapeseed	No probe	-	-	NT
	Sugar cane	No probe		-	No probe

The presence of the target species was ascertained for RT-qPCR Cq values <34. With Cq values between 34 and 35 the species was considered to be present only as traces, and values of Cq>35, which is the cut off limit, associated with no detection. By the end-point methodology we defined the presence or the absence of the same plant species based on visualization of the expected band on the agarose gel electrophoresis. These results are reported on Table 7 (see also S4 Fig) as detected (D) *versus* not detected (ND). In the case of very low band intensity the target species was considered as present in traces.

On general terms and with reference to the species studied, noncompliance to the declared composition was found for almost all feed samples, independently of the methodology used (end-point PCR/ Taqman assays). Unconformities to the label were also detected using TBP methodology applied to feed analysis [11]. Errors or counterfeits related either to missing ingredients, declared in the label but actually absent (flax in CPR-X10 and sunflower in CPR-X13) or, most often, to the presence of non-declared species. The non-declared species can be contaminants (e.g. sunflower in feed CPR-X10) or other species component (e.g. wheat in CPR-X8). Only in the PT1 sample a full compliance with the composition declared in the label was found.

The ingredients compounding a feed provide to cattle the required daily ration of calories and fibers together with protein and fat. These latter materials together with the concomitant presence of potentially harmful anti-metabolites, restrict their amount at few percentages of weight [22,23]. RT-qPCR methodology based on *AOX* genes is providing an important contribute on the detection of these species in complex mixtures.

Discrepancies found by comparing the two approaches are in most cases attributable to the higher sensitivity of the qPCR methodology with respect to end-point PCR. Barley in CPR-X10 and sunflower in CPR-X42, although assumed as present in traces by end-point PCR, actually recorded as not detected by qPCR, because the Cq values were close to the cut-off limit of 35.

### ILC analysis

The ability of the AOX end-point method for feed authentication was tested through an European Inter-laboratory comparison of methods, using the same samples [13]. Here we present the AOX-based RT- results of qPCR experiments performed on those samples and compare them with the results of the end-point methodology (Table 8). To this purpose, seven samples of different composition were prepared starting from eight different raw materials. Laboratory samples (100 g) of each mix were analyzed in our lab. The results are reported on Table 8, together with the nominal values of the seven test samples.

**Table 8:** Results of the ILC test using TaqMan assay and end-point PCR methodology based on *AOX* genes. End-point results are reported in Braglia et al. (2017). TCN: target copy number; D: detected; ND: not detected.

Mix	Species present	%w/w	AOX TaqMan probes		AOX end-point probes (D/traces/ND)
			Cq value	TCN80	
ILC1	Maize	50	25.48	5647.0	D
	Soybean	34.8	26.17	7867.8	D
	Sunflower	8	27.12	30973.5	D
	Flax	7.5	27.56	27999.6	D
ILC2	Durum+soft wheat	45	25.89	18735.3	D
	Maize	12	28.15	859.4	D
	Barley	15	26.99	1749.7	D
	Soybean	18	28.39	1620.1	D
	Rice	10	29.44	312.4	D
ILC3	Barley	100	25.04	7325.6	D
ILC4	Durum+soft wheat	20	28.63	3386.1	D
	Maize	55	27.28	1688.2	D
	Sunflower	10	31.31	2472.8	ND
	Cotton	15	27.91	1972.4	D
ILC5	Durum+soft wheat	100	25.01	41067.8	D
ILC6	Maize	25	27.90	1020.4	D
	Barley	60	25.60	5024.7	D
	Soybean	10	29.03	1117.1	D
	Flax	5	26.73	6790.0	D
ILC7	Durum+soft wheat	20	27.42	8331.2	D
	Maize	20	27.80	1132.7	ND
	Barley	20	26.86	1943.0	D
	Soybean	20	27.83	2389.3	D
	Sunflower	5	29.39	7715.6	D
	Flax	5	26.71	6728.0	D
	Rice	5	31.98	53.2	D
	Cotton	5	28.28	1599.7	D

AOX qPCR methodology provided results always in agreement with the expected qualitative composition of the samples. AOX end-point methodology did not provide accurate results in only two cases (sunflower in ILC4 and maize in ILC7).

## Conclusion

Traditional microscope-based analysis relies on the recognition of the different plant species based on the crushing and the sequential meshing of the feed, possibly combined with staining procedures. Typically, this results in a high level of uncertainty due to a wide range of factors including structure of individual components, methods used in feed manufacture, choice of raw materials and skillness and experience of the analysts. The search for alternative methods, less dependent from human skill and expertise, that can thus be applied in a more systematic and reproducible way, is becoming an urgent need for the implementation of the ever increasing monitoring actions to be carried out on feed and food. DNA-based methods, based on the amplification of a specific DNA fragment, can offer a good alternative for the detection of plant species in complex mixtures. The DNA barcoding technique has been used for recognition of plants but is still problematic in mixtures and cannot support quantitative measurements [24,25]. RT-PCR although requiring expensive equipment, provides an accurate DNA-based method for both recognition and quantitation [2,26]. However, no method based on the identification of DNA of plant origin in compound feeds has been officially recognized by authorities in order to control quality and authenticity of animal feeds. The Feed-Code project, “Animal feed certification instrument and procedure to guarantee the quality of meat and dairy products through automatic, simple and rapid DNA barcode method based on tubulin-based polymorphism (TBP)", financed under the EU FP7 Capacities program, took up the challenge and worked out an effective control system that can properly address the issues raised by the different EC regulations reported above. The TBP (Tubulin-Based-Polymorphism) molecular marker is applied to feed analysis resulting in an easy and efficient DNA profiling of the different botanical species that are actually present in any given feed sample [11]. Here it is shown that AOX-based methodology, initially proposed for validation of the TBP method in the framework of the Feed-Code project, defines an additional suitable molecular marker approach to ascertain plant species composition of animal feed samples. The RT-qPCR technique applied to *AOX* genes shown in this study provides a robust technique for plant species detection at both specificity and sensitivity level.

## Supporting information

S1 Fig Amplification plots to assess primers/probe specificity. Cross-reactivity in gDNA of 31 plant species, bulked into seven pools were performed. The nine plant species selected to design primers and probes were: maize, barley, soybean, wheat, flax, sunflower, peanut, rice and cotton.
(PDF)

S2 Fig Amplification plots from dilution series of gDNA from the selected plant species. gDNA dilutions ranged from final concentration of 0.04 ng to 80 ng, in a total of nine data points. The plant species were: maize, barley, soybean, wheat, flax, sunflower, peanut, rice and cotton.
(PDF)

S3 Fig Sensitivity and linearity of the TaqMan qPCR assays. Serial dilutions of gDNA extracted from sunflower, rice, soybean, cotton, barley, flax, wheat, maize and peanut were tested by using a species-specific TaqMan assay. Data are expressed as the absolute target copy number (TCN) versus gDNA amount. Each data point is the mean of two technical replicates.
(PDF)

S4 Fig Results from end-point methodology applied to 7 feed samples (X8, X10, X13, X32, X42, Z2 and Z3) to identify the presence/absence of 9 plant species. The plant species were wheat, maize, soybean, barley, sunflower, cotton, flax, rice and peanut.
(PDF)

S1 Table List of the 31 species. (DOCX)

S2 Table Composition of the pools used for cross reactivity tests. (DOCX)
