# Supplementary figures and images for: A TaqMan real-time PCR method based on alternative oxidase genes for detection of plant species in animal feed samples
Source: PLoS One. 2018 Jan 2;13(1):e0190668. doi: 10.1371/journal.pone.0190668 (PMC5749836; doi:10.1371/journal.pone.0190668)

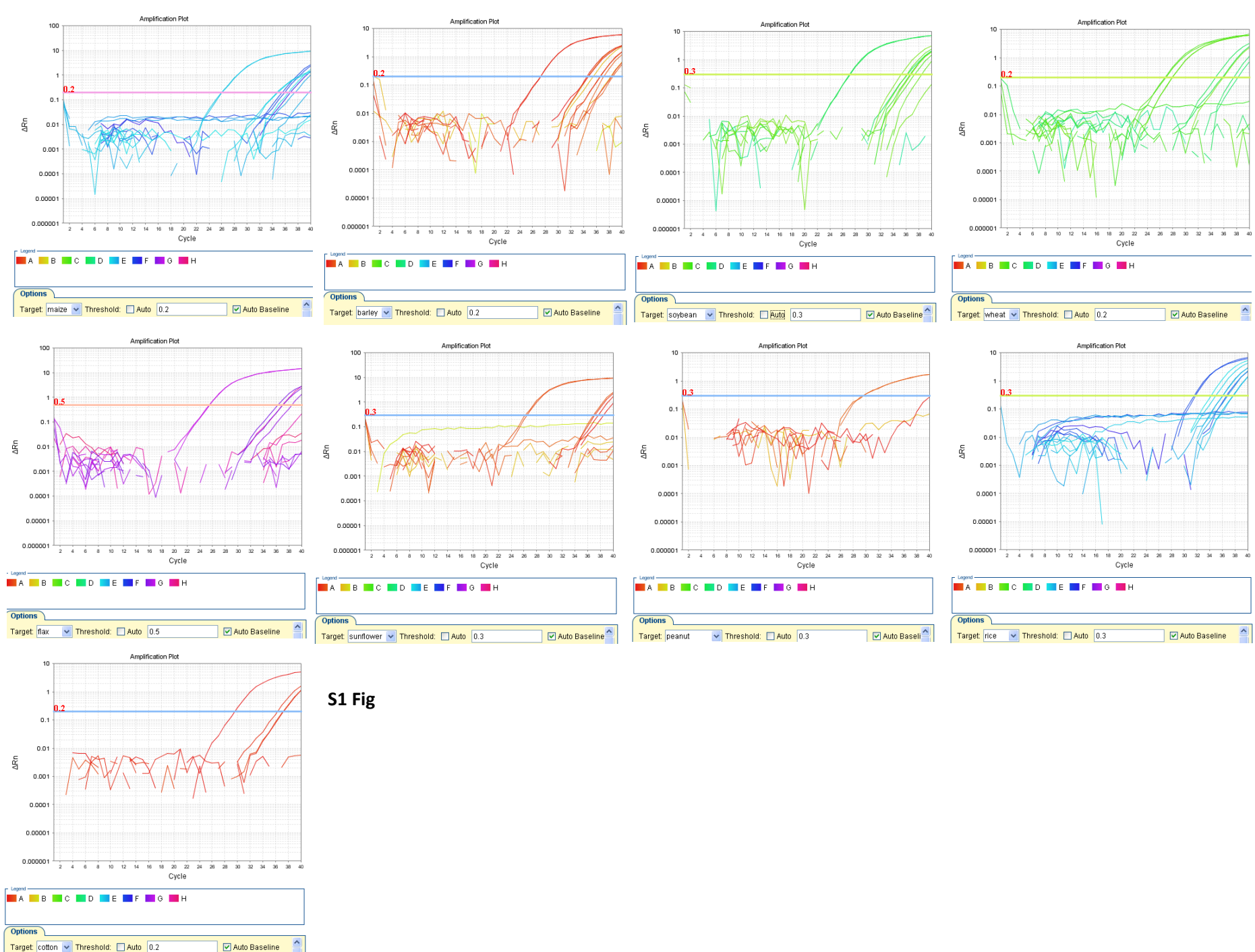

Supplement: S1 Fig — Cross-reactivity in gDNA of 31 plant species, bulked into seven pools were performed. The nine plant species selected to design primers and probes were: maize, barley, soybean, wheat, flax, sunflower, peanut, rice and cotton. (PDF) [file pone.0190668.s001.pdf]

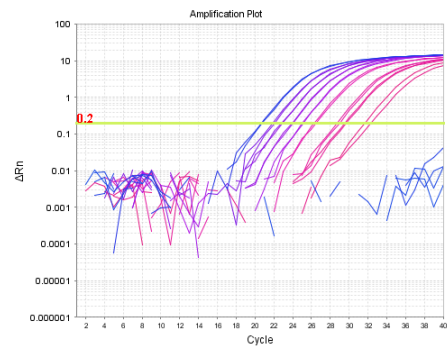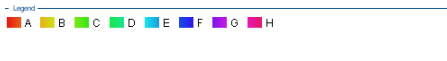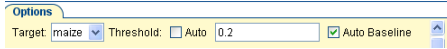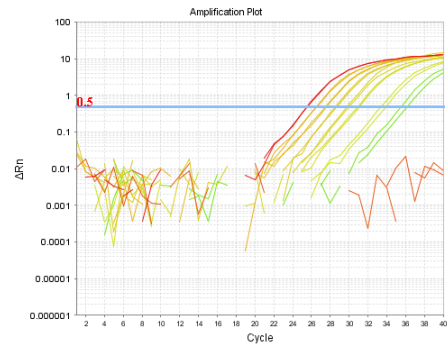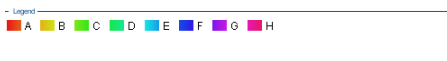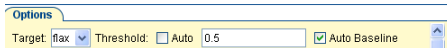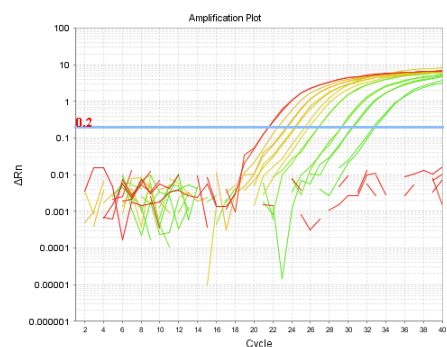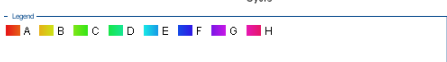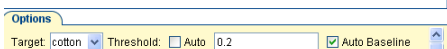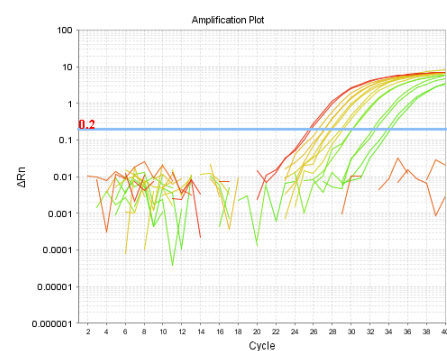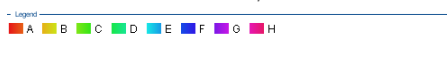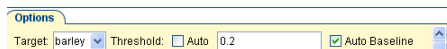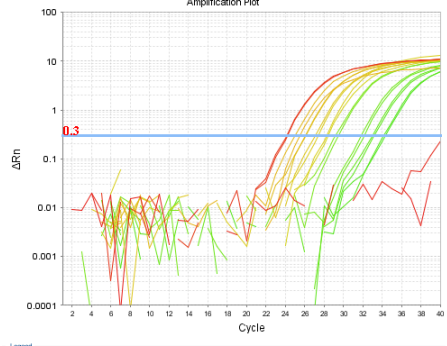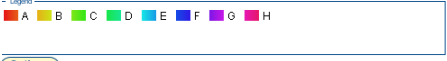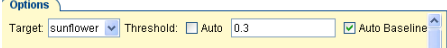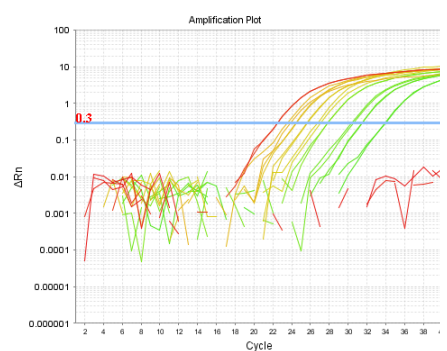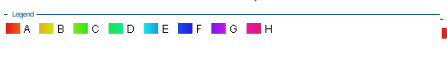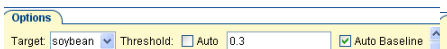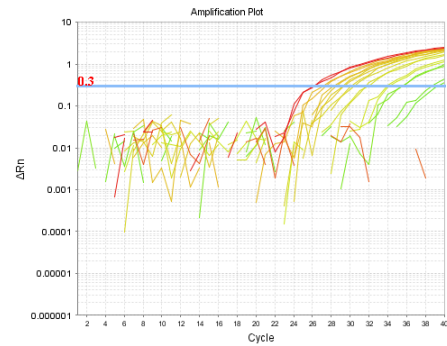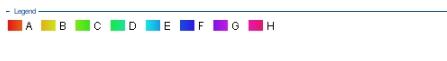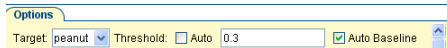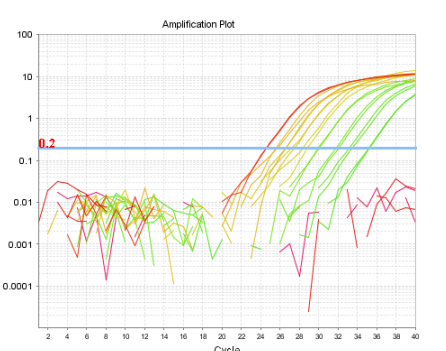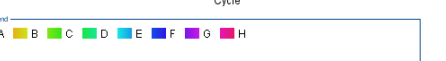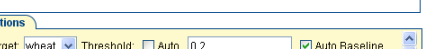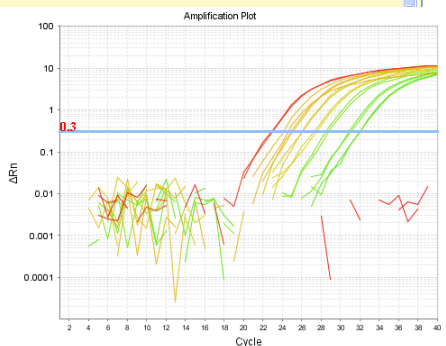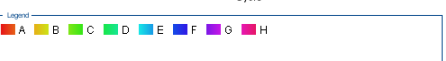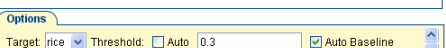

S2 Fig

Supplement: S2 Fig — gDNA dilutions ranged from final concentration of 0.04 ng to 80 ng, in a total of nine data points. The plant species were: maize, barley, soybean, wheat, flax, sunflower, peanut, rice and cotton. (PDF) [file pone.0190668.s002.pdf]

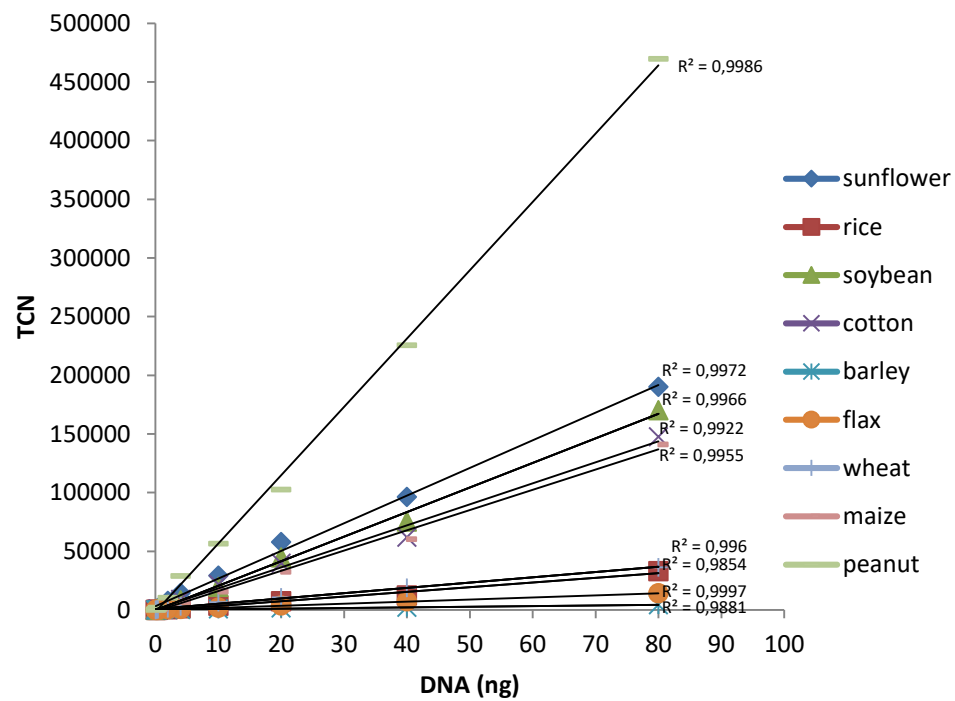

S3\_Fig

Supplement: S3 Fig — Serial dilutions of gDNA extracted from sunflower, rice, soybean, cotton, barley, flax, wheat, maize and peanut were tested by using a species-specific TaqMan assay. Data are expressed as the absolute target copy number (TCN) versus gDNA amount. Each data point is the mean of two technical replicates. (PDF) [file pone.0190668.s003.pdf]

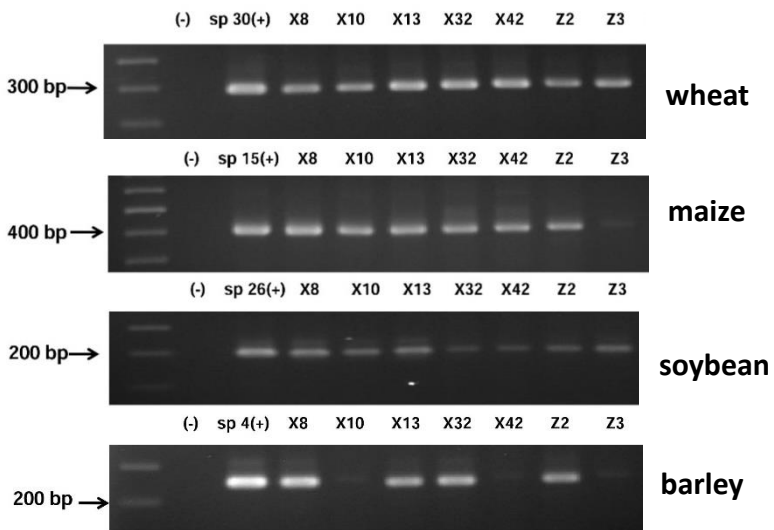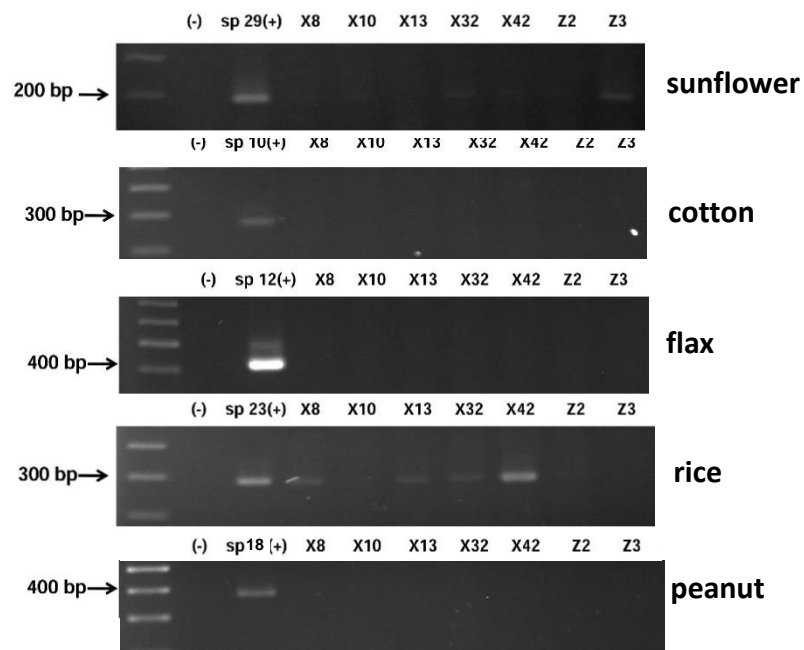

**S4 Fig**

Supplement: S4 Fig — The plant species were wheat, maize, soybean, barley, sunflower, cotton, flax, rice and peanut. (PDF) [file pone.0190668.s004.pdf]
