# Supplementary material for: A TaqMan real-time PCR method based on alternative oxidase genes for detection of plant species in animal feed samples
Source: PLoS One. 2018 Jan 2;13(1):e0190668. doi: 10.1371/journal.pone.0190668 (PMC5749836; doi:10.1371/journal.pone.0190668)
Supplement: S1 Table — (DOCX) [file pone.0190668.s005.docx]

**S1 Table** List of the 31 species.

|  | **Common name** | **Latin name** | **Cultivar** |
| --- | --- | --- | --- |
| **1** | Alfalfa | *Medicago sativa* L. | ND |
| **2** | Apple | *Malus domestica* Borkh | Golden Delicious |
| **3** | Barley | *Hordeum vulgare* L. | ND |
| **4** | Beetroot | *Beta vulgaris* L*. convar. Crassa* var. *conditiva* | Rossa Detroit |
| **5** | Carrot | *Daucus carota* L. | Berlicum |
| **6** | European Chestnut | *Castanea sativa* Miller | Castagna di Montella |
| **7** | Clover (red) | *Trifolium pratense* L. | Nike |
| **8** | Clover (white) | *Trifolium repens* L. | ND |
| **9** | Common Vetch | *Vicia sativa* L. var. *sativa* | ND |
| **10** | Cotton | *Gossypium hirsutum* | ND |
| **11** | Field beans | *Vicia fava* L. | ND |
| **12** | Flax | *Linum usitatissimum* L. | Valoal |
| **13** | Hazelnut | *Corylus spp* L. | Nocciola di Giffoni |
| **14** | Lentil | *Lens culinaris* L. | ND |
| **15** | Maize | *Zea mays ssp. mays* | Indentata |
| **16** | Millet | *Panicum miliaceum* L. | ND |
| **17** | Oat | *Avena sativa* L. | ND |
| **18** | Peanut | *Arachis hypogaea* L. | ND |
| **19** | Feed Pea | *Pisum sativum subsp. arvense* (L.) Asch | ND |
| **20** | Potato | *Solanum tuberosum* L. | Kennebec |
| **21** | Pumpkin | *Cucurbita maxima Duch* | ND |
| **22** | Rapeseed | *Brassica napus* L. ssp. *Napus* | Rossini |
| **23** | Rice | *Oryza sativa* L. | Fino R.I.B.E. |
| **24** | Rye | *Secale cereal* L. | ND |
| **25** | Sorghum | *Sorghum bicolor* (L.) *Moench.* | Bravis |
| **26** | Soybean | *Glycine max* L*.* Merr | ND |
| **27** | Spelt | *Triticum spelta* L. | ND |
| **28** | Sugar beet | *Beta vulgaris* L. var. *saccharifera* | Verdi |
| **29** | Sunflower | *Helianthus annuus* L. | ND |
| **30** | Wheat (durum) | *Triticum durum* Desf | VAL group |
| **31** | Wheat (soft) | *Triticum aestivum* L. | ND |

ND: Not determined
