# Supplementary material for: A TaqMan real-time PCR method based on alternative oxidase genes for detection of plant species in animal feed samples
Source: PLoS One. 2018 Jan 2;13(1):e0190668. doi: 10.1371/journal.pone.0190668 (PMC5749836; doi:10.1371/journal.pone.0190668)
Supplement: S2 Table — (DOCX) [file pone.0190668.s006.docx]

**S2 Table**

| **Pool 1** | **Pool 2** | **Pool 3** | **Pool 4** | **Pool 5** | **Pool 6** | **Pool 7** |
| --- | --- | --- | --- | --- | --- | --- |
| Cotton | Sorghum | Carrot | Spelt | Apple | Peanut | Hazelnut |
| Rye | Field beans | Maize | Beetroot | Common Vetch | Potato | Soybean |
| Millet | Barley | Wheat (soft) | Lentil | Sunflower | Chestnut | Clover (white) |
| Rapeseed | Feed Pea | Oat | Alfalfa | Pumpkin | Flax | Clover (red) |
|  |  | Wheat (durum) | Sugar beat |  |  | Rice |
